# Knowledge, attitude and practice of adolescent girls towards reproductive health: a cross-sectional study in Turkistan region, Kazakhstan

**DOI:** 10.2144/fsoa-2022-0054

**Published:** 2023-03-13

**Authors:** Kenzhegul Ryskeldiyeva, Ikilas Moldaliyev, Saule Tuktibaeva, Raushan Nurkhassimova, Saule Kurbaniyazova, Aigerim Kushkarova, Sandugash Ramanova

**Affiliations:** 1 Khoja Akhmet Yassawi International Kazakh-Turkish University, Turkistan, Kazakhstan; 2 Department of Obstetrics & Gynecology, Khoja Akhmet Yassawi International Kazakh-Turkish University, Turkistan, Kazakhstan; 3 Ecomed Turkistan Clinic, Doctor reproductololgy Center, Turkistan, Kazakhstan

**Keywords:** adolescent girls, menstruation, reproductive health, social status

## Abstract

**Background:**

Reproductive health of adolescent females is an important health concern.

**Aim:**

To determine the impact and the knowledge, attitude and practice of female adolescents toward reproductive health.

**Materials & methods:**

This is a survey based cross-sectional study conducted in Turkistan region.

**Results:**

A total of 1250 participants were included with a mean age of 17.3 ± 1.4 years, and >80% of the participants have completed high school. A total of 1191 girls had the onset of menarche at approximately 13.2 years old, and 85.7% reported menstrual disorder.

**Conclusion:**

There is poor knowledge and practice of reproductive health among participating adolescents. Alcohol consumption, high BMI, bad family relationships and lack of gynecological visits were found to negatively affect reproductive health.

## Plain language summary

Improving adolescent girl’s knowledge toward reproductive health can help them make better and informed reproductive health choices. We studied a sample of adolescent girls in Turkistan region in Kazakhstan and found that the majority of the surveyed girls have poor reproductive health. Several issues were found to negatively influence reproductive health of adolescent girls including obesity (measured by BMI), consumption of alcohol, lack of visit of reproductive healthcare experts and strained family relationships.

Menarche is the first menstrual cycle that reflects the sexual and reproductive development of females, and it is one of the most noticeable developmental events during adolescence years that occurs between the ages of 10 and 16 years [1]. However, the average age for the onset of menarche depends on several factors including nutrition, environmental conditions and socio-economic status of the individual [2,3]. Also, the formation and regularity of the menstrual cycle are considered as important indicators for reproductive health of childbearing females including adolescents [4]. However, menstrual disorders including dysmenorrhea, menorrhagia, hypomenorrhea, oligomenorrhea and dysfunctional uterine bleeding are commonly reported during adolescence years [5,6]. These diseases are influenced by biological, environmental and social factors [1,2,4]. For the latter, studies suggested that the family’s socioeconomic background not only affect the adolescents’ behavior, but it can significantly impact their reproductive health as well [7]. The sexual and reproductive health of adolescents are closely associated with their social, cultural and economic environments [2,3,7]. Adolescents in low and middle-income countries are at a significantly high risk of acquiring sexual transmitted diseases [8].

In Kazakhstan, a developing central Asian country with a total population of 19 million (as of 2021) of which approximately 15% adolescent girls, the incident rates of gynecological diseases among adolescents during 2014–2019, were reportedly increased by more than 50% [9]. However, these reports did not investigate contributing or associated factors. Nevertheless, adolescence is a period of rapid growth and sexual exploration, during which they encounter several challenges particularly in the developing world [4]. The UN Reproductive Health Agency, reported that adolescent girls in developing countries are highly vulnerable and likely to have early pregnancy, violence, HIV and less likely to have access to sexual and reproductive healthcare including contraception and professional help during pregnancy and childbirth [9]. Thus, an adolescent with good reproductive knowledge would likely make the right decision, as the outcome of the choices they take during this period would likely to influence their future.

Therefore, it is quite important to determine the adolescents’ knowledge attitude and practice toward reproductive health and the impact of the socioeconomic backgrounds on the reproductive health of adolescent girls in Kazakhstan. Thus, the current study aims to determine the impact of adolescents’ knowledge, attitude and practice toward reproductive health. The study will also determine the impact of socioeconomic status of their families on their reproductive health. We hypothesized that determining the adolescents’ knowledge, attitude and practice and their socioeconomic status will allow us to improve reproductive healthcare services for this important population.

## Materials & methods

### Study setting & design

This is a cross-sectional questionnaire-based study that was conducted in Turkistan, which is one of most ethnically diverse regions in Kazakhstan with limited infrastructures and availability of healthcare services. The study was conducted from October 2021 until March 2022. The questionnaire used in the current study was adapted from Herdman *et al.* and Pleasant *et al.*, that were developed to assess knowledge of reproductive health and the impact of socioeconomic status [10,11]. The questionnaire contains 32 questions divided into different sections; including a section about the participant’s demographic data and other sections designed to test the participant’s knowledge, practice and attitude toward reproductive health.

The menarche age was calculated retrospectively and recorded by age in years. Height and weight were measured according to the protocol described by *Martin* *et al.* [12]. Briefly, the height and weight were measured with an anthropometer to the nearest 0.1 cm, and the nearest 0.1 kg, respectively. BMI (kg/m^2^) was determined and used as an indicator of healthy weight status of the participants. The questionnaire was validated with a pilot run using a group of 20 randomly selected individuals (13 adolescent girls (10–15 year old) and seven young adults (16–25 year old) who were surveyed to ensure reliability and suitability of the survey. The results of the pilot test indicated minor changes and based on the results of the pilot run, the final corrected version of the questionnaire was used to execute the current study.

### Study population (inclusion/exclusion)

Participants were recruited by convenience sampling method from four different secondary schools in Turkistan region (two urban and two rural schools) and three special educational colleges (Turkistan Higher Medicine and Turkistan Multidisciplinary Medicine College, Turkistan Pedagogical College) and first and second year University students of Khoja Akhmet Yassawi. For participants under the age of 18 years, a parental or guardian consent was requested. The parents/guardians of these individuals as well as all participants were made aware that this study is for research purposes only and their participation was voluntary. They were not asked for their names, email address or contact information, ensuring the privacy of survey respondents. Those who are under 18 years of age without adult consents, and the over 18 year old who did not consent to participate were excluded from the study.

### Data storage

All data collection forms were kept in a secure setting, only available to the principal investigator.

### Statistical analysis

Data were recorded on a data collection form and entered on a Microsoft Office Excel^®^ (2013) spreadsheet. The statistical analyses were performed using SPSS Statistics 24. The Shapiro–Wilk and Kolmogorov–Smirnov tests were performed as normal distribution tests. Student’s *t*-test was used to compare two independent groups suitable for normal distribution, and the Mann–Whitney U test was used in paired groups not suitable for normal distribution. Chi-square test was used to compare categorical variables. A p-value of less than 0.05 was considered statistically significant.

## Results

### Response rate

A total of 1500 questionnaires distributed to cover for the required participants at 95% CI, plus 10% attrition rate for non respondents or incomplete questionnaires. There was 1300 completed surveys, 50 of which were excluded (invalid/incomplete responses) bringing the survey response rate to 83.3%.

### Demographic data

The mean age of the participating girls was 17.3 ± 1.4 years, including 39.4% identified as college students, 37.8% were university students, high school students 17.5% and students with higher education 5.4% (Table 1).

**Table 1.** Characteristics of the participants.

Variables	Mean ± SD	Percentage
Age	17.3 ± 1.4	100%
Educational background College student University student High school student Higher education	492 472 219 67	39.4% 37.8% 17.5% 5.4%
BMI Height (cm) Weight (Kg)	162.4 ± 8.3 53.8 ± 8.9	
Menses Yes No	1191 59	95.3% 4.7%
Average age of menarche	13.2 ± 1.2	
Average duration of menses (days)	4.8 ± 1.4	
Normal menses	181	14.48%
Menses disorders	1071	85.7%
Delayed Irregular Painful Others	330 345 306 90	30.8% 32.2% 28.6% 8.4%

### Socioeconomic status

The average parental age for the participants fathers was 48.8 ± 6.6, and for their mothers was 44.8 ± 5.7. Out of the respondents, 56.7% live in their own homes, 23.9% in rented apartments and 19.4% in dormitories. The average number of children in the family was 6.1 ± 1.8, including 2.3 ± 1.3 girls. The results are showing in Table 2. While the employment rate for parents (both fulltime and self-employed parents) was estimated to be 57.4% for fathers and 56% for mothers, there was a significantly high percentage of parental unemployment (44% mothers and 42.6% fathers). The high unemployment rate could be due to the recent pandemic as the study was performed during this period. Thus, in order to further determine the socioeconomic status of the participants, we included a direct financial questions (i.e., availability of money in the house). For example, those who selected ‘always’ for lack of money in the family, were considered to have a low socioeconomic background; those who selected ‘sometime’ were designated as middle-class; and those who selected ‘never’ were considered to have high socioeconomic status. In total, 12.3% of the participants were considered to be from low socioeconomic background, 56.8% were designated as middle-class, and the remaining 30.9% were considered to have high socioeconomic status (Table 2).

**Table 2.** Socioeconomic indicators.

Family status	Mean ± SD	Percentage
Live with both parents	1041	83.3%
Live with single parent	209	16.7%
Parents		
Average age: father	48.8 ± 6.6	
Employment status		
Working (including own business)	718	57.4%
Not working	532	
Average age: mother	44.8 ± 5.7	
Employment status		
Working (including own business)	700	56%
Not working	550	44%
Family own home	709	56.7%
Rented apartment	299	23.9%
Dormitory	242	19.4%
Number of family members	6.1 ± 1.8	
Number of girls in the family	2.3 ± 1.3	
Lack of money		
Always Sometimes Never	154 710 386	12.3% 56.8% 30.9%
Family relationship		
Good Normal Bad	932 249 69	74.6% 19.9% 5.5%
Smoking Yes No	46 1204	3.7% 96.3%
Alcohol Yes No	70 1180	5.6%% 94.4%
Diet		
Healthy balanced diet Imbalanced high meat diet Unhealthy diet	656 464 130	52.5% 37.1% 10.4%

### Age of menses

While 59 girls (4.7%) did not yet menstruate, the remaining 1191 (95.3%) of adolescent girls did. The mean age of the onset of menarche was 13.2 ± 1.2 years old, and menstruation duration of 4.8 ± 1.4 days (Table 1). The average height is 162.4 ± 8.3 cm and the average weight is 53.8 ± 8.9 kg. Compared with the 181 (14.48%) girls who reported normal menstruation, the majority of the girls 85.7% (1071 girls) reported various menstrual disorders with irregular, delayed or painful menstruation as being the most commonly reported disorders (Table 1).

Furthermore, to determine the volume of menstruation, the girls were asked to select the number of menstrual pads that they use during each period, after which they were divided into different groups. For example, low menstrual volume group (oligo) included girls who use 1 pad per day, normal menstrual volume group included those who use 2–5 pads per day and for those who use six or more pads per day, included in the high menstrual volume group (hyper). Interestingly, there was a significant association between menstrual disorders and body weight and family relationship (Table 3).

**Table 3.** Menstrual disorders and modifiable risk factors.

Variable	β (95% CI)	p-value
Menses disorders (reference all disorders)	0.566 (0.506, 0.491)	0.151
Normal BMI (18.5–24.9 kg/m^2^) vs high BMI (25–29.9 kg/m^2^)	0.361 (0.230, 0.344)	<0.001^†^
Good family relationship vs bad	0.271 (0.485, 0.592)	<0.001^†^
Healthy diet vs unhealthy diet	0.402 (0.320, 0.314)	<0.12
Gynecological visits vs no gynecological visits	0.512 (0.385, 0.377)	<0.05^†^
Alcohol consumption vs no alcohol	0.371 (0.674, 0.661)	<0.03^†^

Multivariable adjusted for menses disorders, BMI and smoking.

† Indicate significance.

### Health status

**Figure 1.**
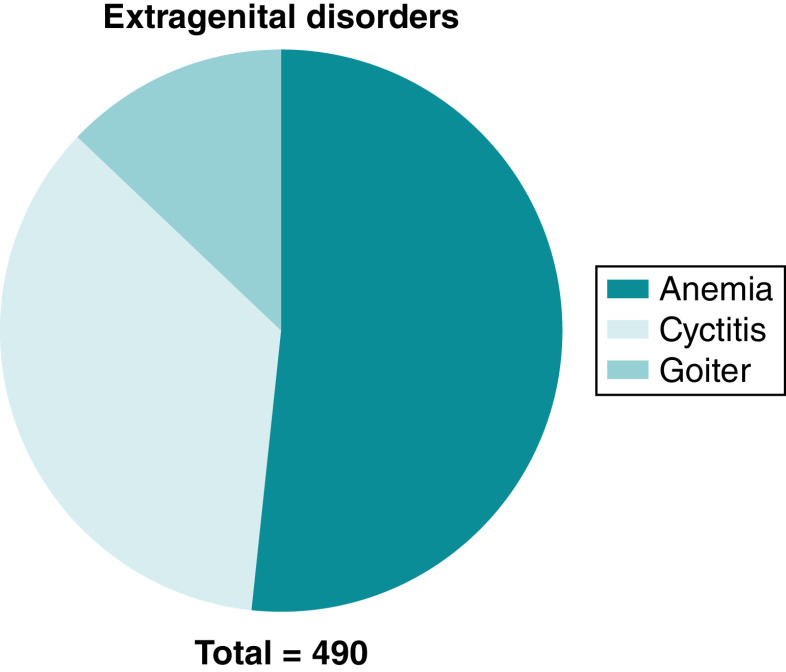
Extragenital disorders.

In addition to reproductive health, the participants were surveyed about general health status. Out of the total surveyed participants, 490 girls (39.2%), indicated that they have existing extragenital disorders with anemia as the most recorded disorder among the participants (20.2%), followed by cystitis (13.9%) and goiter, which was reported in about 5% of the participants (Figure 1).

### Knowledge of reproductive health

In order to determine the participants knowledge of reproductive health, the participants were asked about their knowledge of sexual transmitted diseases and the different types of contraceptives. The results show that the majority of the participating girls have low knowledge of sexually transmitted diseases (96.1%) and the different types of contraceptives or their measures (96.8%), (Table 4). These figures indicate an alarming low level of reproductive literacy and knowledge among the participating girls.

**Table 4.** Respondent’s knowledge and attitude toward reproductive health.

Item	Percentage
Knowledge of sexual transmitted diseases	3.9%
Knowledge of types of contraceptives	3.2%
Believe in gynecological testing	75.1%
Seek gynecological help	17.7%
Life style is not related to reproductive health	63%

### Practice & attitude toward reproductive health

One of the most important indicators of health practice and attitude is dietary intake. Interestingly, the majority of the girls (52.5%), reported to mostly consume a healthy balanced diet that contains adequate meat, fruits, vegetables and dairy products; 37.1% reported to consume an imbalanced high meat diet; and the remainder 10.4% reported to commonly consume unhealthy diet (Table 2). The participants were asked whether they seek professional help from gynecologists or not for reproductive health issues. While, the vast majority of the participants (75.1%) believe that gynocological testing is important for reproductive health, more than 1000 participants do not seek gynecological help 82.3% if they have a reproductive issue (Table 4). In addition, we asked the participants to report other unhealthy or risky habits including smoking and alcohol drinking. The vast majority 94.4% do not consume alcohol, and 96.3% do not smoke tobacco (Table 2). However, alcohol consumption was strongly associated with poor reproductive knowledge and that alcohol consumption is associated with menstrual disorders (Table 3). Despite believing in the importance of gynecological testing, multivariant analysis showed that girls who do not seek gynecological assistant were likely to have low reproductive knowledge and have one form of menstrual disorder (Table 3).

## Discussion

The current study investigated the knowledge, attitude and practice of adolescent girls living in Turkistan region, southern of Kazakhstan toward reproductive health. The study included 1250 participants from different schools and institutes in the region including more than 80% have completed at least 12 years of study (high school). The mean age of the participants was 17.3 years of whom, 1191 had the onset of menarche at the average age of 13.2 years old and the remaining 59 participants did not yet have their menarche. Interestingly, the vast majority of the participants (85.7%) reported some sort of menstrual disorders. This is a very high percentage, and due to the lack of similar studies or published statistics in the country, we cannot compare the results locally. However, internationally the prevalence was significantly different between countries, for instance, an Australian study that investigated menstrual patterns and disorders in Australian teenagers, claimed that 25% of the participants reported menstrual disorder [13]. Whereas, an Indian study from rural areas in Tamil Nadu, reported menstrual disorders in close to 90% of the participants [14]. The variation is, of course, due to several variable and nonvariable factors [2,13,15].

However, the results showed poor knowledge and practice of reproductive health among adolescent girls measured by their knowledge of sexual transmitted diseases and types of contraceptives. The impact of poor knowledge of reproductive health can be reflected by the high percentage (>85%) of participants who reported different types of menstrual disorders. The results are similar to previous reports from developing countries including, Hamadanieh and colleagues who conducted a cross-sectional study to assess the sexual and reproductive knowledge of women in Lebanon [16]. They reported that less than 10% of the participants to have adequate reproductive health knowledge. Similarly, a study by Kyilleh *et al.*, that assessed adolescents’ reproductive health knowledge, reported a significantly low level of reproductive health knowledge among the participating adolescents that was negatively affecting their reproductive health [17].

While the present results showed no correlation between diet and reproductive health, BMI was identified as a strong factor that could influence menstrual disorders. For instance, girls with BMI of 25–29.9 kg/m^2^ were likely to experience reproductive health issues. This appears to be a widely accepted factor with several studies supported this finding including, Chavarro and colleagues who studied the impact of nutrition on reproductive health. The authors claimed that a BMI value of more than 25 kg/m^2^ or less than 19 kg/m^2^ could negatively affect women’s reproductive health [18]. Another widely accepted confounder that negatively affects reproductive health was alcohol consumption. In this study, the results showed that alcohol consumption is closely associated with reproductive health, and that girls who consume alcohol significantly increase their risk of developing menstrual disorders. This finding is in line with several studies that suggest alcohol consumption to impact reproductive health, disrupt female puberty and that the impact will significantly increase if consumed during puberty period [19]. Interestingly, girls who have bad family relationship were the most likely participants to smoke tobacco or consume alcohol. Family relationship was also indicated as a significant factor associated menstrual disorders. For example, girls with bad family relationship were likely to report menstrual disorders. This may perhaps be due to stress, as it has been shown to negatively influence reproductive health [16,20].

Nevertheless, more than 60% of the participants believe that lifestyle does not affect reproductive health. Although, we did not investigate the reasons for this, we can speculate that this may in part be due to the significantly poor knowledge of reproductive health. While, more than three-quarters of the participants believe/trust gynecological testing, less than 18% seek or visit a gynecology if they experience a reproductive health issue. This may partly be due to the lack of available gynecological services in Turkistan, as well as the poor practice and attitude of the participants’ toward reproductive health.

## Study limitation

This study is a cross-sectional study that can show association, but does not reflect the causal relationship. Also, the study was conducted in one region (Turkistan) only. While it is a large and ethnically diverse region, the results do not necessary reflect the general population. However, the results can provide insights for future health promotion and intervention of adolescent reproductive health.

## Conclusion

In conclusion, this is the first study to investigate reproductive health in the adolescent population in Kazakhstan. The present study shows the majority of the participants suffer some form of menstrual disorder that were associated with poor knowledge of reproductive health, alcohol consumption, high BMI and bad family relationship. It also showed the importance of gynecological assessment of adolescents to improve their reproductive health. Thus, the results of the current study highlight the need of pediatric gynecological services in the region. Thus, it is recommended that the Turkistan regional health authority, with the support of the Ministry of Health and the participation of different medical specialties, must initiate a region-wide gynecological services for the adolescent population. It is also important to increase the awareness of reproductive health among adolescents through health campaigns or inclusion in school curricula.

**Summary points**

- Adolescence is a phase of growth and development.
- In developing world, adolescent girls are vulnerable due to lack of reproductive knowledge.
- Improving adolescent girl’s knowledge toward reproductive health can improve future choices.
- A cross sectional study conducted in Turkistan region, southern of Kazakhstan.
- Participants recruited by convenience sampling from different locations.
- A total of 1250 completed surveys were collected and analyzed.
- The mean age of the participants was 17.3 ± 1.4 and nearly 40% are college students.
- Some of the participants reported extragenital with anemia as the most commonly reported.
- An overall poor knowledge and practice of reproductive health.
- The majority of the participants reported some sort of menstrual disorders.
- Poor knowledge and practice of reproductive health appears to be associate with reproductive disorders.
- Other factors such as BMI, alcoholic consumption and lack of family support are associated with poor reproductive health knowledge and practice.
